# Supplementary material for: Cryopreservation impairs 3-D migration and cytotoxicity of natural killer cells
Source: Nat Commun. 2020 Oct 16;11:5224. doi: 10.1038/s41467-020-19094-0 (PMC7568558; doi:10.1038/s41467-020-19094-0)
Supplement: Supplementary file 1 — Supplementary Information [file 41467_2020_19094_MOESM1_ESM.pdf]

Supplementary Information

Cryopreservation impairs 3-D migration and cytotoxicity of natural killer cells

Mark et al.

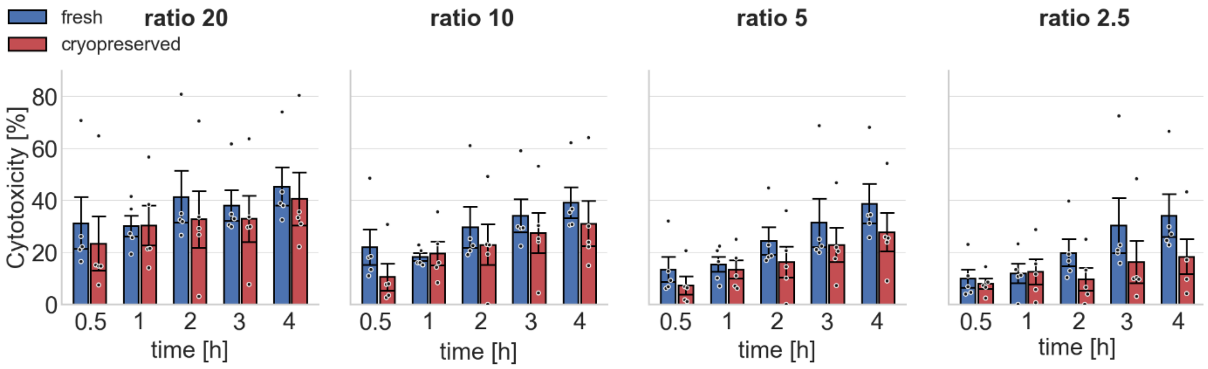

Supplementary Figure 1: Cytotoxicity (mean ± se) of 5 independent isolations (4 subjects, 3 different expansions) for fresh (blue) and cryopreserved (red) NK cells with different effector to target ratio (20:1,10:1,5:1,2.5:1) at different times (0.5h, 1h, 2h, 3h, 4h). At shorter incubation times (0.5 and 1 h) and low NK-to-target ratio, the cytotoxicity between fresh and cryopreserved cells equalizes. Error bars denote 1 SEM. Source data are provided as a Source Data file.

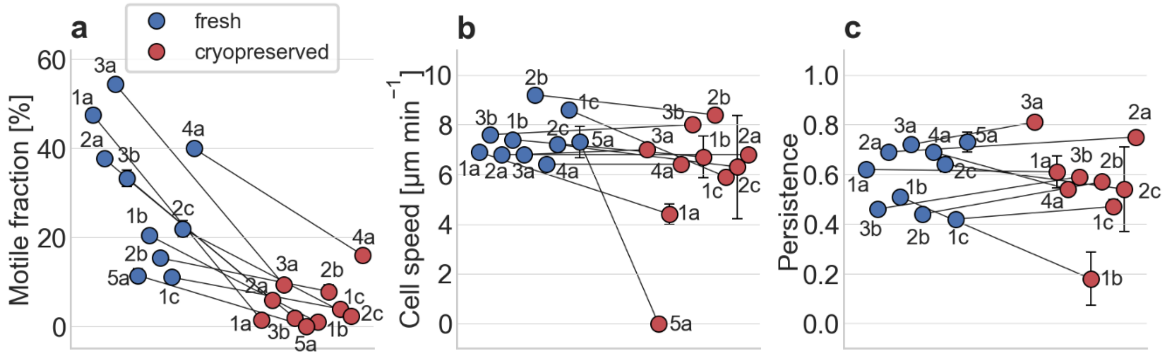

Supplementary Figure 2: Motile fraction, migration cell speed and migration persistence of fresh (blue) and cryopreserved (red) NK cells from n = 10 independent experiments from 5 subjects (1-5) and different expansion (a,b,c) in 1.2 mg/ml collagen. Each symbol represents mean ± se from cells measured in 5 fields of view, with approximately 80 cells (motile plus non-motile) in each field of view. Migration speed and persistence is computed from on average of n = 125 motile fresh cells and n = 12 motile cryopreserved cells for each subject and expansion (each data point). In total, n = 1248 motile fresh and n = 122 motile cryopreserved NK cells are measured. Paired (fresh versus cryopreserved) data from each subject and expansion are connected by lines. **a:** Motile fraction. **b:** Cell speed. **c:** Directional persistence. Source data are provided as a Source Data file.

* indicates a measurement where not a single NK cell in 5 fields of view was migratory. Therefore, mean cell speed and mean directional persistence could not be determined.

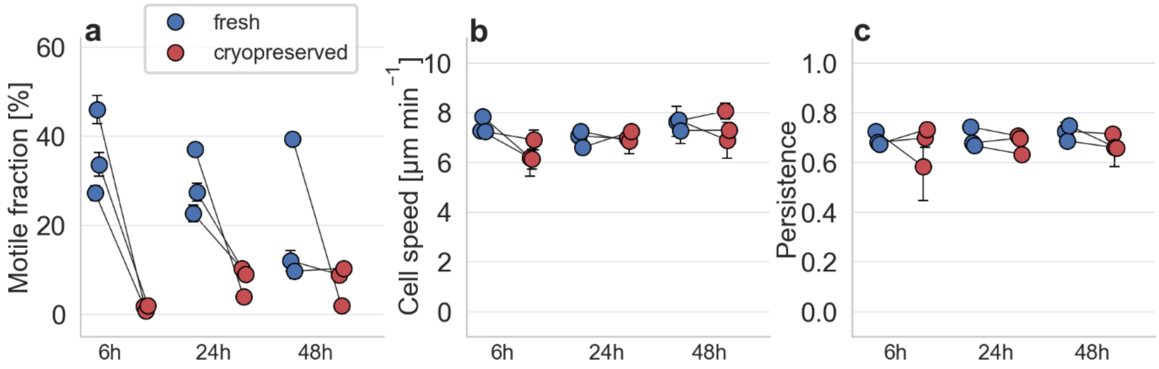

Supplementary Figure 3: Motile fraction, migration cell speed and migration persistence of fresh (blue) and cryopreserved (red) NK cells from n = 3 independent isolations (3 subjects) in 1.2 mg/ml collagen for different incubation times in RPMI 1640 medium (2h, 20h, 44h). After expansion or thawing, cells are mixed in collagen solution after 2h (= 6h measurement) in medium, after 20h in medium, and after 44h in medium. After 1 hour of gel polymerization we incubate the samples for three more hours so that measurements start 6h, 24h and 48h after thawing or expansion. Each symbol represents mean ± se from 10 fields of view for each subject, with approximately 80 cells in each field of view. Paired (fresh versus cryopreserved) data from each subject are connected by lines. Data for migration speed and persistence are based on in total n = 1508 motile fresh and n = 628 motile cryopreserved NK cells. **a:** Motile fraction **b:** Cell speed **c:** Directional persistence. Source data are provided as a Source Data file.

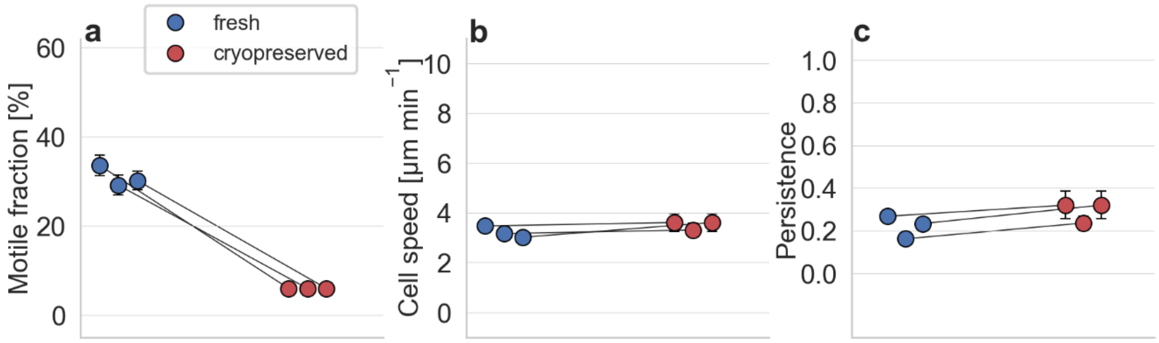

Supplementary Figure 4: Motile fraction, migration cell speed and migration persistence of NK92 cells (ATCC CRL-2407) from n=3 independent experiments in 1.2 mg/ml collagen. NK92 cells are grown in Alpha MEM without nucleosides with 15% FCS, 15 HS and 500 IU/ml IL-2. Cryopreservation and thawing is performed as for ex-vivo expanded NK cells (see Methods). Each symbol represents mean ± se from 10 fields of view for each independent experiment, with approximately 80 cells in each field of view. Paired (fresh versus cryopreserved) data from each independent experiment are connected by lines. Data for migration speed and persistence are based on in total n = 650 motile fresh (blue) and n = 125 motile cryopreserved (red) NK92 cells. **a:** Motile fraction **b:** Cell speed **c:** Directional persistence. Source data are provided as a Source Data file.

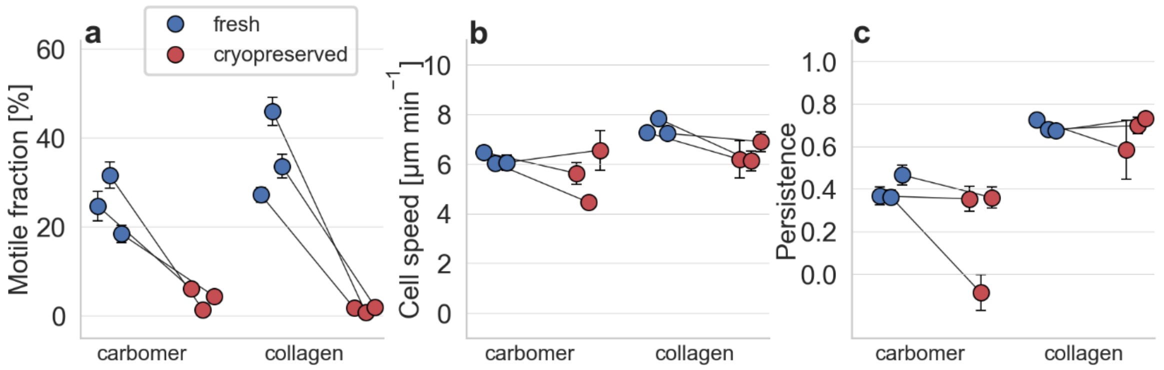

Supplementary Figure 5: Motile fraction, migration speed and migration persistence of fresh (blue) and cryopreserved (red) NK cells from n=3 independent isolations (3 subjects) in non-adhesive copolymer hydrogels of acrylic acid and alkyl-methacrylate (9mg/ml carbomer) and in 1.2 mg/ml collagen. Each symbol represents mean ± se from 10 fields of view for each subject, with approximately 75 cells in each field of view. Paired (fresh versus cryopreserved) data from each subject are connected by lines. Data for migration speed and persistence are based on in total n = 657 motile fresh and n = 125 motile cryopreserved NK cells. **a:** Motile fraction **b:** Cell speed **c:** Directional persistence. Source data are provided as a Source Data file.

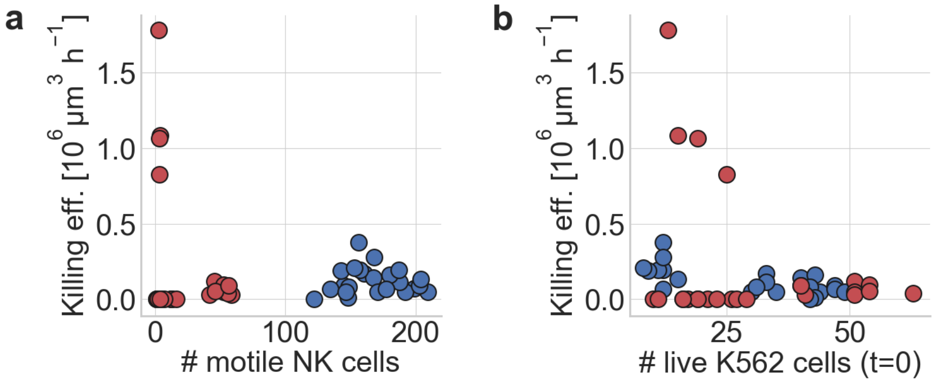

Supplementary Figure 6: a: Killing efficiency for individual fields-of-view as a function of the number of motile fresh (blue) and motile cryopreserved (red) NK cells as determined by the 3-D cytotoxicity assay (same donors and expansions as in Fig. 4 in the main text). **b:** Killing rate for individual fields-of-view as a function of the number of K562 target cells. We find no systematic dependence of the killing rate on either the number of NK cells or the number of target cells, demonstrating that the killing rate estimation is not biased by different concentrations of NK cells and target cells. Source data are provided as a Source Data file.

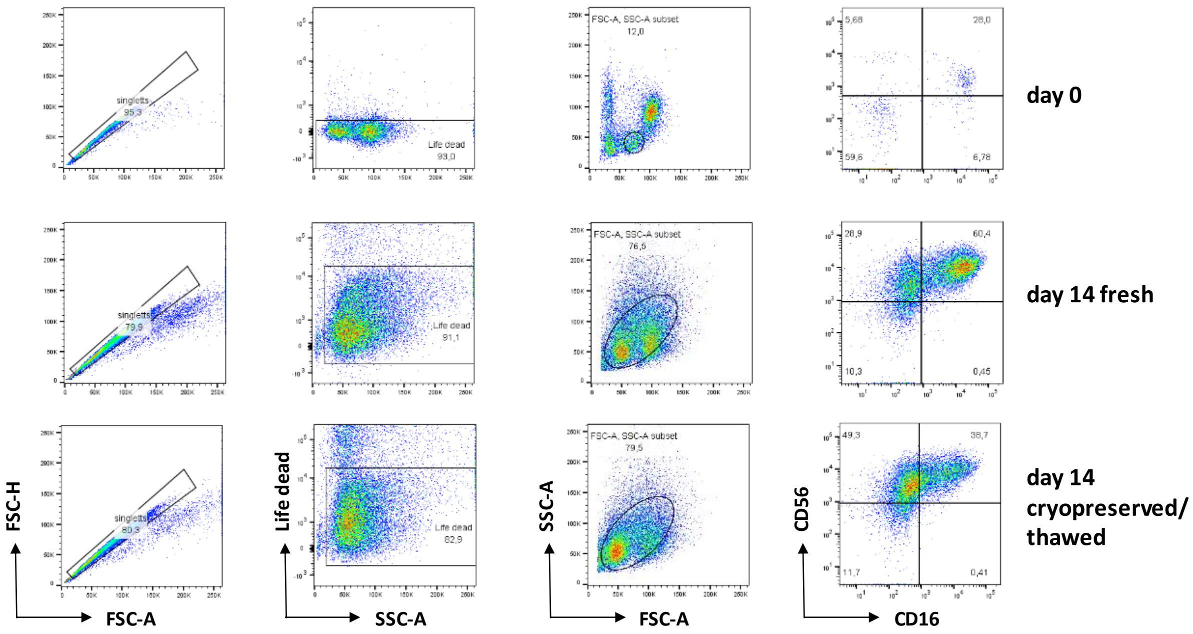

Supplementary Figure 7: Cells are first gated for singlets (FSC-H versus FSC-A) and life cells (Life dead versus SSC-A). Next, lymphocytes are gated based on size and granularity by forward and side scatter (SSC-A versus FCS-A). Finally, the lymphocyte gate is analyzed for expression of NK cell-specific markers (e.g. CD3, CD56, CD16, CD107a).

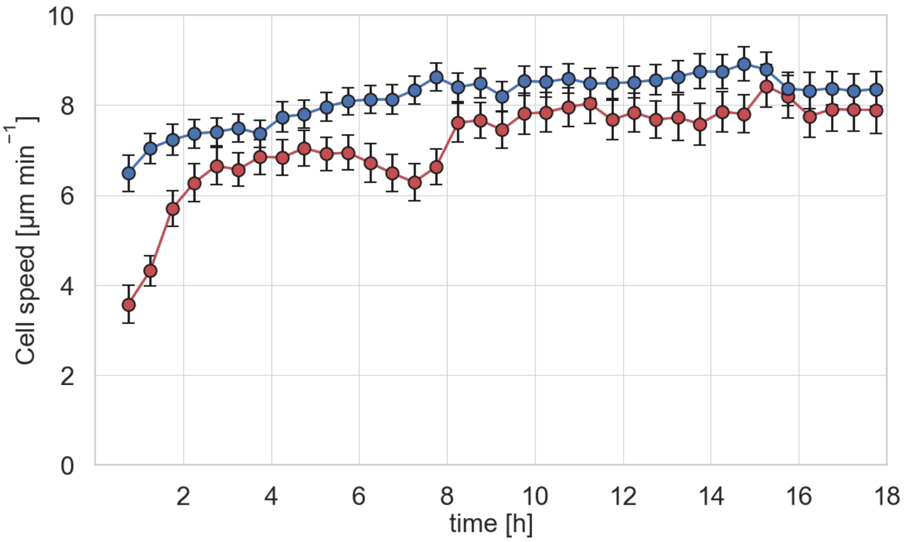

Supplementary Figure 8: Recovery of cell speed after time in collagen for fresh (blue) and cryopreserved (red) NK cells from 3 different subjects. Measurement of cell migration started immediately following the completion of the collagen polymerization process 60 min after embedding the cells. For computing the mean cell speed, only the motile fraction of the cells is analyzed. Cells can migrate into and out of the microscope's field of view so that the number of cells from which the mean cell speed is computed fluctuates over time. On average, the mean value is computed from 115 motile fresh cells (blue circles) and 105 motile cryopreserved cells (red circles). Error bars denote 1 SEM. Source data are provided as a Source Data file.

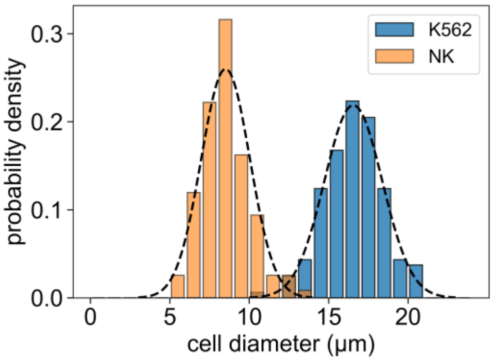

Supplementary Figure 9: Size distribution of K562 cells (blue) and non-motile (round) NK cells (orange) (K562 n = 162, NK n = 117). The overlap in the size distributions is less than 2%. Source data are provided as a Source Data file.

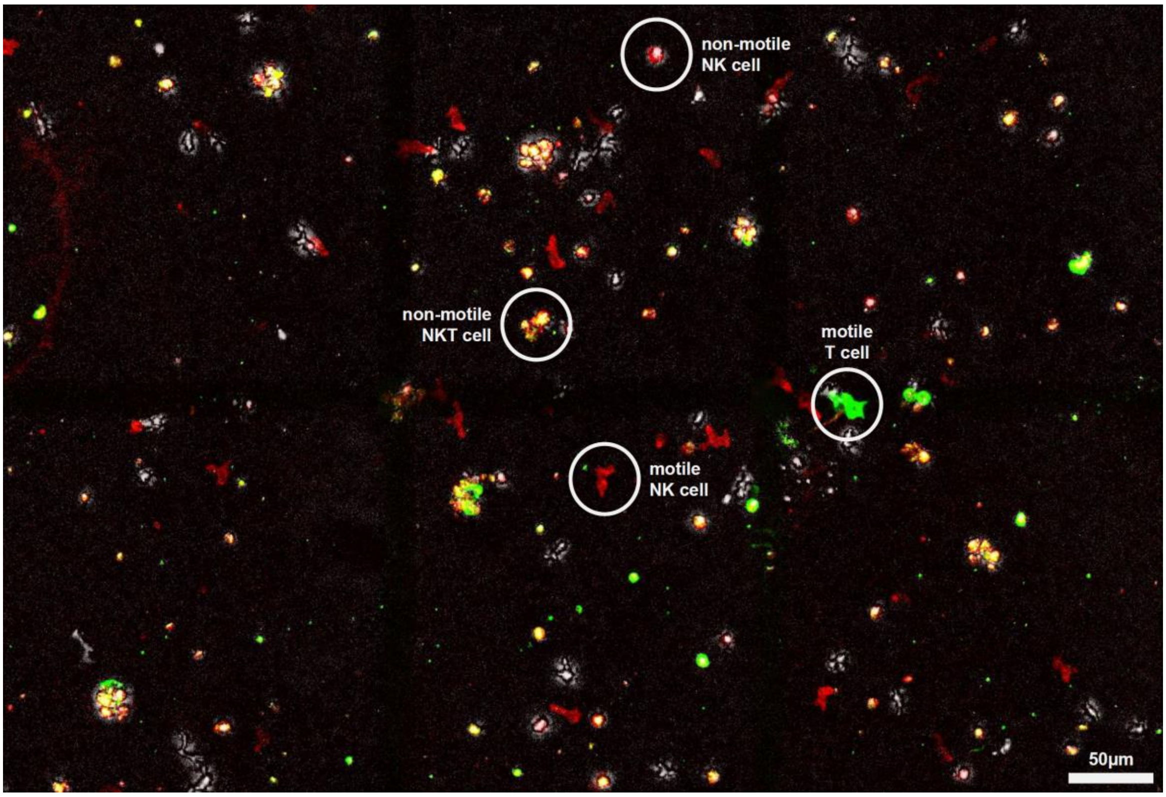

Supplementary Figure 10: Composite confocal and bright-field maximum intensity projections of a cell population expanded from PBMCs in a 3D collagen gel. The brightfield maximum intensity projection at the beginning of the experiment is shown in greyscale. The red overlay shows the confocal maximum intensity projection of a CD56-APC staining that was recorded 10 minutes after recording the brightfield image stack. The green overlay shows the confocal maximum intensity projection of a CD3-Alexa488 staining that was also recorded 10 minutes after recording the brightfield image stack. Thus, cells that appear red on a dark background are motile NK cells, while cells that appear red on a bright background have not moved during the last 10 minutes and represent non-motile NK cells. Cells that appear yellow (double positive for CD56 and CD3) are NKT cells, and cells that appear green are T cells. Based on three field-of-views, we find that 82% of all motile cells are NK cells. The experiment was set up similar to the 3D cell motility assay described in the Methods section and was carried out once.

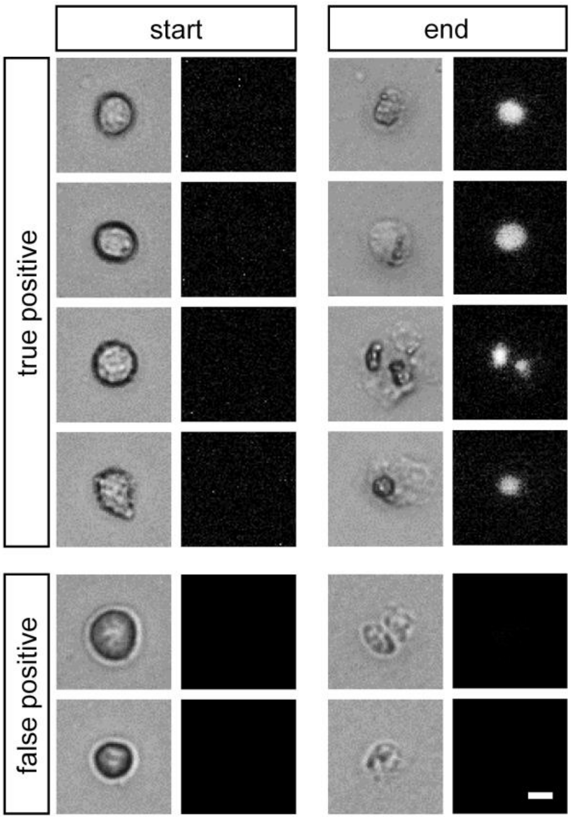

Supplementary Figure 11: Killing events as evaluated based on bright field images are verified with NucRed Dead 647 staining (Ready Probes, Thermo Fisher). 89.4% of all K562 cells that are classified as “dead” based on bright field criteria are stained positive (true positives). 10.6% of all K562 cells that are classified as “dead” based on bright field criteria are stained negative (false positives). These cells may possibly be undergoing apoptosis but may still have an intact cell membrane. In n=196 evaluated cells, we found no false negative events (no cell that was classified as “living” stained positive). Scale bar: 10 µm.
