# Supplementary material for: Cryopreservation impairs 3-D migration and cytotoxicity of natural killer cells
Source: Nat Commun. 2020 Oct 16;11:5224. doi: 10.1038/s41467-020-19094-0 (PMC7568558; doi:10.1038/s41467-020-19094-0)
Supplement: Supplementary file 7 — Reporting Summary [file 41467_2020_19094_MOESM7_ESM.pdf]

nature research

Corresponding author(s): Ben Fabry
Last updated by author(s): Sep 5, 2020

Reporting Summary

Nature Research wishes to improve the reproducibility of the work that we publish. This form provides structure for consistency and transparency in reporting. For further information on Nature Research policies, see Authors & Referees and the Editorial Policy Checklist.

Statistics

For all statistical analyses, confirm that the following items are present in the figure legend, table legend, main text, or Methods section.

n/a	Confirmed	
☐	☒	The exact sample size (n) for each experimental group/condition, given as a discrete number and unit of measurement
☐	☒	A statement on whether measurements were taken from distinct samples or whether the same sample was measured repeatedly
☐	☒	The statistical test(s) used AND whether they are one- or two-sided<br>*Only common tests should be described solely by name; describe more complex techniques in the Methods section.*
☒	☐	A description of all covariates tested
☐	☒	A description of any assumptions or corrections, such as tests of normality and adjustment for multiple comparisons
☐	☒	A full description of the statistical parameters including central tendency (e.g. means) or other basic estimates (e.g. regression coefficient) AND variation (e.g. standard deviation) or associated estimates of uncertainty (e.g. confidence intervals)
☐	☒	For null hypothesis testing, the test statistic (e.g. F, t, r) with confidence intervals, effect sizes, degrees of freedom and P value noted<br>*Give P values as exact values whenever suitable.*
☒	☐	For Bayesian analysis, information on the choice of priors and Markov chain Monte Carlo settings
☒	☐	For hierarchical and complex designs, identification of the appropriate level for tests and full reporting of outcomes
☒	☐	Estimates of effect sizes (e.g. Cohen's d, Pearson's r), indicating how they were calculated

Our web collection on statistics for biologists contains articles on many of the points above.

Software and code

Policy information about availability of computer code

Data collection	Images were collected with a in-house developed Python program that controls the motorized microscope stage via a standard serial connection and that uses the open-source interface library µManager 2.0-gamma (micro-manager.org) to control the camera .
Data analysis	Images were analyzed with in-house developed programs based on Python 3.7. Specifically, image segementation for cell detection was carried out using the open-source Deep Learning framework Tensorflow 1.15 (tensorflow.org), the U-Net architecture (which is available as open-source code for different Deep Learning frameworks on github.com), and the image annotation software ClickPoints 1.8.0. The algorithm for cell tracking is described in detail in the Methods section of the mansucript. Statistical analysis was carried out using RStudio 1.2.5019 (rstudio.com) and the open-source Python library SciPy 1.3.0 (scipy.org). Flow cytometry data was analyzed using FlowJo V10.

For manuscripts utilizing custom algorithms or software that are central to the research but not yet described in published literature, software must be made available to editors/reviewers. We strongly encourage code deposition in a community repository (e.g. GitHub). See the Nature Research guidelines for submitting code & software for further information.

Data

Policy information about availability of data

All manuscripts must include a data availability statement. This statement should provide the following information, where applicable:

- Accession codes, unique identifiers, or web links for publicly available datasets
- A list of figures that have associated raw data
- A description of any restrictions on data availability

The datasets generated during and/or analyzed during the current study are available from the corresponding author on request.

Field-specific reporting

Please select the one below that is the best fit for your research. If you are not sure, read the appropriate sections before making your selection.

☒ Life sciences ☐ Behavioural & social sciences ☐ Ecological, evolutionary & environmental sciences

For a reference copy of the document with all sections, see nature.com/documents/nr-reporting-summary-flat.pdf

Life sciences study design

All studies must disclose on these points even when the disclosure is negative.

Sample size	Based on the availability of patient samples and the complexity of the involved experiments, cells derived from 11 subjects and 17 independent expansions were investigated (resulting in 24 expansion/donor combinations; samples from more than one patients may be handled in parallel during one expansion process). Additional experiments requested by the reviewers confirmed initially submitted results. We ensured that the variability between cell donors and between individual cell expansions was considered in the study by collecting data from multiple donors and multiple expansions from individual donors. Regarding the number of individual cells that were measured in each experiment, we strived for the largest technically feasible number by imaging multiple field-of-views in parallel, giving us data from typically > 500 cells for each donor or cell expansion.
Data exclusions	No data was excluded in experiments that were deemed successful.
Replication	The findings generated from donor-derived cells were replicated for multiple cell donors and multiple cell expansions for individual donors. Furthermore, the findings were confirmed by experiments using an established cell line that corresponds to the cell type of the donor-derived cells.<br><br>Cell viability measurements were performed independently 9 times with fresh and cryopreserved NK cells, respectively, based on NK cells from 4 different donors and for 8 different expansions.<br><br>Cell population specification, chromium release assay, and 3D cell motility assay were performed independently 10 times with 5 different donors and for 5 different expansions.<br><br>The degranulation assay was performed independently 8 times with 4 different donors and for 4 different expansions.<br><br>The 3D cytotoxicity assay was performed independently 6 times, with 4 different donors and for 3 different expansions.<br><br>The additional chromium-release cytotoxicity experiments shown in the Supplementary Information were performed independently 5 times with 4 different donors and 3 different expansions.<br><br>The additional 3D cell motility experiments shown in the Supplementary Information were performed independently 3 times with 3 different donors and a single expansion.<br><br>The 3D cell motility experiments using the NK92 cell line shown in the Supplementary Information were performed independently 3 times.
Randomization	Randomization was not necessary as the investigators performing the cell experiments had no further information on the cell donors. The study did not aim to correlate findings from cell experiments with medical records from cell donors but investigated general properties of donor-derived cells.
Blinding	Investigators performing the cell experiments had no further information on the cell donors.

Reporting for specific materials, systems and methods

We require information from authors about some types of materials, experimental systems and methods used in many studies. Here, indicate whether each material, system or method listed is relevant to your study. If you are not sure if a list item applies to your research, read the appropriate section before selecting a response.

Materials & experimental systems

n/a	Involved in the study
☐	☒ Antibodies
☐	☒ Eukaryotic cell lines
☒	☐ Palaeontology
☒	☐ Animals and other organisms
☐	☒ Human research participants
☒	☐ Clinical data

Methods

n/a	Involved in the study
☒	☐ ChIP-seq
☐	☒ Flow cytometry
☒	☐ MRI-based neuroimaging

Antibodies

Antibodies used	CD3 (clone: UCHT1; supplier: Biolegend)<br>CD16 (clone: 3G8; supplier: Biolegend)<br>CD56 (clone: HCD56; supplier: Biolegend)<br>CD107a (clone H4A3; supplier: BD Biosciences)
Validation	All antibodies were used according to the manufacturer's instructions. Validation statements by the supplier:<br><br>CD3: "Each lot of this antibody is quality control tested by immunofluorescent staining with flow cytometric analysis and the oligomer sequence is confirmed by sequencing."<br><br>CD16: "Each lot of this antibody is quality control tested by immunofluorescent staining with flow cytometric analysis."<br><br>CD56: "Each lot of this antibody is quality control tested by immunofluorescent staining with flow cytometric analysis."

Eukaryotic cell lines

Policy information about cell lines

Cell line source(s)	- K562-mbIL15-41BBL feeder cells: gift from Prof. D. Campana, Department of Pediatrics, University Hospital Singapore; formerly St. Jude Children's Research Hospital, Memphis, TN, USA<br>- NK-92 cell line: purchase from ATCC CRL-2407
Authentication	NK-92 cell line came authenticated following purchase from ATCC. K562-mbIL15-41BBL feeder cells have been previously published and validated. No additional authentication procedures were conducted.
Mycoplasma contamination	K562 feeder cells and NK92 cells are confirmed negative for mycoplasma contamination using the Venor GeM Classic detection kit (Minerva Biolabs).
Commonly misidentified lines<br>(See ICLAC register)	No commonly misidentified cell lines were used.

Human research participants

Policy information about studies involving human research participants

Population characteristics	Healthy males and females, aged 18-65 years, who approved to donate their leukoreduction system chamber (LRSC). A LRSC is a left-over product from a routine donor plateletpheresis procedure.
Recruitment	Donors were asked for their permission to donate their LRSC as part of the informed consent for a donor plateletpheresis procedure. The primary reason of the donors to undergo a plateletpheresis is the willingness to donate platelets for transfusion purposes outside the scope of our study (e.g. for treatment of patients with clotting disorders). In general, the platelet donors are asked to donate their left-over LRSC for scientific puposes, including our study. The distribution of the LRSC is random among the scientific projects requiring them. Overall, platelet donors represent the healthy general population without any bias.
Ethics oversight	Ethics Commission of the Friedrich-Alexander University Erlangen-Nürnberg (IRB approval number 147_13B)

Note that full information on the approval of the study protocol must also be provided in the manuscript.

Flow Cytometry

Plots

Confirm that:

☒ The axis labels state the marker and fluorochrome used (e.g. CD4-FITC).

☒ The axis scales are clearly visible. Include numbers along axes only for bottom left plot of group (a 'group' is an analysis of identical markers).

☒ All plots are contour plots with outliers or pseudocolor plots.

☒ A numerical value for number of cells or percentage (with statistics) is provided.

Methodology

Sample preparation	Human blood samples (LRSC) were otained from the Department of Transfusion Medicine (University Hospital Erlangen) and whole peripheral blood mononuclear cells (PBMC) were isolated using density-gradient centrifugation. Next, NK cells were expanded from PBMC as previously described. In brief, PBMC (1.5 × 10e6) were incubated with irradiated (14,000 rad) K562-mbIL15-41BBL cells (10e6) in a 24-well tissue culture plate in the presence of 200 IU/ml human IL-2 in RPMI supplemented with fetal calf serum, L-Glutamine and gentamycin (herafter called cRPMI). Half of the culture medium was replaced every 2-3 days

with fresh culture medium for the first 7 days. After 7 days of expansion, cells are harvested, washed, and re-cultured in T-25 or T-75 culture flasks in cRPMI supplemented with IL-2.

Instrument	BD Canto II flow cytometer (BD Biosciences)
Software	Flowjo (FLOWJO, LLC Data analysis software)
Cell population abundance	No cell sorting was performed in this study
Gating strategy	Cells were first gated for singlets (FSC-H versus FSC-A) and life cells (Life dead versus SSC-A). Next, lymphocytes were gated based on size and granularity by forward and side scatter (SSC-A versus FCS-A). Finally, the lymphocyte gate is analyzed for expression of NK cell-specific markers (e.g. CD3, CD56, CD16, CD107a).

☒ Tick this box to confirm that a figure exemplifying the gating strategy is provided in the Supplementary Information.
